# Influence of Ink Properties on the Morphology of Long-Wave Infrared HgSe Quantum Dot Films

**DOI:** 10.3390/nano12132180

**Published:** 2022-06-24

**Authors:** Suhui Wang, Xu Zhang, Yi Wang, Tengxiao Guo, Shuya Cao

**Affiliations:** State Key Laboratory of NBC Protection for Civilian, Beijing 102205, China; wangsuhui1995@163.com (S.W.); m4a1hitman@sina.com (X.Z.); wangyi102205@sina.com (Y.W.)

**Keywords:** HgSe QD, long-wave infrared, evaporated film, morphology

## Abstract

As the core device of the miniature quantum dot (QD) spectrometer, the morphology control of the filter film array cannot be ignored. We eliminated strong interference from additives on the spectrum of a long-wave infrared (LWIR) QD filter film by selecting volatile additives. This work is significant for detecting targets by spectroscopic methods. In this work, a filter film with characteristic spectral bands located in the LWIR was obtained by the natural evaporation of QD ink, which was prepared by mixing various volatile organic solvents with HgSe QD–toluene solution. The factors affecting the morphology of HgSe LWIR films, including ink surface tension, particle size, and solute volume fraction, were the main focus of the analysis. The experimental results suggested that the film slipped in the evaporation process, and the multilayer annular deposition formed when the surface tension of the ink was no more than 24.86 mN/m. The “coffee ring” and the multilayer annular deposition essentially disappeared when the solute particles were larger than 188.11 nm. QDs in the film were accumulated, and a “gully” morphology appeared when the solute volume fraction was greater than 0.1. In addition, both the increase rate of the film height and the decrease rate of the transmission slowed down. The relationship between film height and transmission was obtained by fitting, and the curve conformed to the Lambert–Beer law. Therefore, a uniform and flat film without “coffee rings” can be prepared by adjusting the surface tension, particle size, and volume fraction. This method could provide an empirical method for the preparation of LWIR QD filter film arrays.

## 1. Introduction

Nanomaterial inkjet printing technology is a cutting-edge technology for the micro-distribution and precise printing of ink droplets by controlling the nozzle voltage, air pressure, platform temperature, and motion trajectory, which can achieve the high-precision patterned deposition of nanomaterials. This technology has attracted extensive attention in the fields of display panel printing [1,2,3,4], microelectronic component fabrication [5,6,7], and flexible printing [8,9,10] in recent years due to the advantages of rapidity, convenience, and low cost.

As is known to all, semiconductor QDs are synthetic nanomaterials. When the size of a semiconductor quantum dot is smaller than or comparable to the exciton Bohr radius in all three directions, the electron motion is confined and forms a split energy level [11,12,13,14,15,16,17,18,19,20,21]. Therefore, it has many unique optoelectronic properties different from those of bulk materials, such as broad absorption spectra, narrow symmetrical emission spectra, and large Stokes shifts. Its spectrum can be tuned by adjusting various parameters, such as the synthesis time, material ratio, and core–shell structure [22,23]. In addition to the QD’s unique optical properties, it also has the advantages of low cost, multiple types, and easy integration. Therefore, people have used QDs as filter materials to prepare visible-light (Vis) and near-infrared (NIR) filter film arrays and QD micro-spectrometers [24,25,26,27,28,29]. The existing filter films and their working bands are shown in Table 1.

Using a QD filter film array prepared by inkjet printing technology as the spectroscopic element of a micro-spectrometer is an effective method to miniaturize the spectrometer. The uniformity of the filter film directly determines the error when the detector detects the light intensity and affects the performance of the spectrometer. Therefore, it is necessary to control the film morphology. There are many factors that affect the morphology of thin films, and they are usually divided into external environmental factors and internal characteristic factors. External environmental factors mainly include ambient temperature, substrate temperature, substrate roughness, droplet size, etc., while internal characteristic factors refer to the ink characteristics, including ink surface tension, solute particle size, concentration, viscosity, etc. In terms of ink conditioning, surfactants or adhesives are usually added to regulate the surface tension of the ink solvent. The effect of capillary flow on particles can be overcome by triggering a tension gradient (Marangoni flow) [30] in droplets [31,32,33,34,35], so a uniform and flat film without “coffee rings” can be obtained [36]. Sometimes, increasing the particle size or changing the particle shape can reduce the effect of capillary force and weaken the “coffee ring” effect [37,38,39]. The solute volume fraction can also be varied to adjust the degree of sparsity or density on deposition patterns [40,41]. As mentioned above, optimizing the film morphology by adjusting the properties of the solvent has excellent effects in printing display panels and preparing visible-light or near-infrared filter films.

The characteristic spectral peak of HgSe QDs used in this study was located in the long-wave infrared at 12.5 μm [42]. The ink cannot be modified simply by adding active agents or polymers when preparing a long-wave infrared filter film. Because most surfactants or adhesives are difficult to evaporate and have strong absorption in the long-wave infrared band, the specific filtering function will not be achieved. Therefore, volatile organic solvents were used to modify the ink in this study. The effects of ink surface tension, particle agglomeration, and the solute volume fraction on the morphology of nanomaterials were investigated.

Eight kinds of evaporable organic solvents (isopropanol, n-octane, ethanol, ethyl acetate, butyl acetate, acetone, chloroform, and toluene) were used as surface tension modifiers. QD inks with different surface tensions were prepared by mixing the organic with toluene–QD solution (toluene was used to ensure the dispersion of QDs). Due to the large difference in polarity between n-octane and toluene, the agglomeration degree of QDs can be regulated by adding n-octane to the QD solution. QD inks with different agglomerated particle sizes could be obtained by mixing different proportions of n-octane and toluene–QD solution. QD inks with different solute volume fractions were prepared by mixing different proportions of toluene and toluene–QD solution. Then, 0.5 μL of the QD solution was dropped on a glass slide with a pipette, simulating the situation of ink droplets on the substrate in inkjet printing. The effects of the surface tension, particle size, and solute volume fraction of the ink solvent on the film morphology were analyzed. The fitting curve of the relationship between the solute volume fraction and transmittance was obtained. The results can provide a reference for the preparation of long-wave infrared QD filter films with specific transmittance and good morphology.

## 2. Materials and Methods

The materials and devices used in our experiments are as follows.

The characteristic absorption peak of the QD that we used is 12.5 μm. The QD solution was prepared by dissolving 50 mg of HgSe QDs in 1 mL of toluene. A 50 mg/mL HgSe QD–toluene solution was used as the solute, and isopropanol, n-octane, ethanol, ethyl acetate, butyl acetate, acetone, chloroform, and toluene were used as different solvents. Eight kinds of QD inks with different solvents were obtained by mixing the solute with solvents in a 1:1 volume ratio. Then, the inks were put into an ultrasonic instrument (Kunshan KQ-50B, Beijing, China) and shaken for 10 min. The surface tension of the inks was measured by an automatic surface tensiometer (Zhongchen POWEREACH, Shanghai, China) with the platinum plate method at 11.2 °C. Then, 0.5 μL of QD inks were dropped by pipette on glass slides, and the films were observed by using an optical microscope (Mingmei ML31, Guangzhou, China) after the solvent evaporated naturally.

The HgSe QD solution was used as the solute, and the solvent was prepared by mixing toluene and n-octane in volume ratios of 5:5, 4:6, 3:7, 2:8, and 1:9, followed by ultrasonic vibration for 10 min. After that, the inks were obtained by mixing the solvents and the solute with ultrasonic vibration for 10 min. The sampling amount is shown in Table 2.

The surface tension of the inks was measured by an automatic surface tensiometer. Then, 0.5 μL of QD inks was dropped by pipette on glass slides. The films were observed using an optical microscope after the solvent evaporated naturally. The area and number of the particles and the “coffee ring” width were counted and measured using the measurement mode.

The HgSe QD solution was used as the solute, and toluene was used as the solvent. Eight kinds of QD inks with volume fractions *φ_μ_* of 0.01, 0.025, 0.05, 0.075, 0.1, 0.25, 0.5, and 0.75 were obtained by mixing different volumes of toluene with the solute (the effect of QD volume on solute volume was ignored in the calculation), followed by ultrasonic vibration for 10 min, where *φ_μ_* = *V_a_*/(*V_a_* + *V_b_*), *V_a_* is the volume of QD solution, and *V_b_* is the volume of toluene. Then, 0.5 μL of QD inks was dropped by pipette on glass slides, and the films were observed using an optical microscope and atomic force microscope after the solvent evaporated naturally. Next, 0.5 μL of QD ink was dropped on the ZnSe window using a pipette. The infrared absorption spectrum of the film was measured using a Fourier transform infrared spectrometer (Thermo iS50 FT-IR, Beijing, China) after the solvent evaporated naturally.

All substrates in the experiments were washed three times with acetone, ethanol, and distilled water sequentially and dried in a vacuum desiccator (DZF-6050, Beijing, China).

## 3. Results

### 3.1. Influence of Ink Surface Tension on FILM Morphology

**Figure 1:**
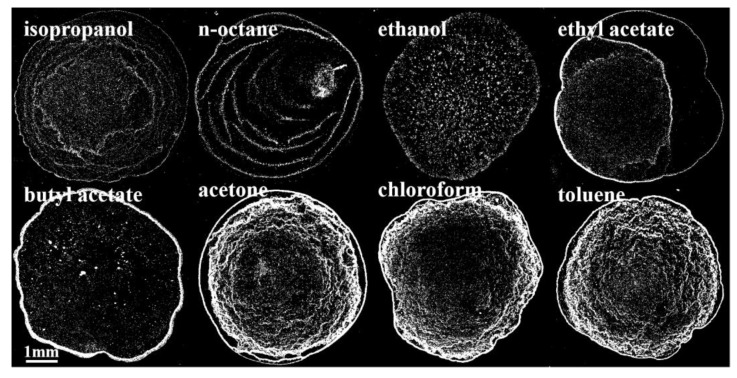
Morphologies of films prepared with different QD inks.

The morphologies of films prepared with eight different solvent inks as observed under an optical microscope are shown in Figure 1.

It can be seen in Figure 1 that the morphologies of the films prepared with QD inks with different solvents are different, but they can be roughly divided into three types: the first type is multilayer annular deposition (isopropanol and n-octane as solvents); the second type is nonspecific deposition (ethanol, ethyl acetate, and butyl acetate as solvents); and the third type is “gully” deposition (acetone, chloroform, and toluene as solvents). Then, the ink properties were measured and calculated to explore the reasons for film formation.

The surface tension $\gamma_{g–l}$ of the ink and the droplet radius *R* were measured, and the contact angle θ of the droplet, the work of adhesion $W_{a}$, the work of immersion $W_{i}$, and the spreading coefficient S were calculated, as shown in Table 3.

**Figure 2:**
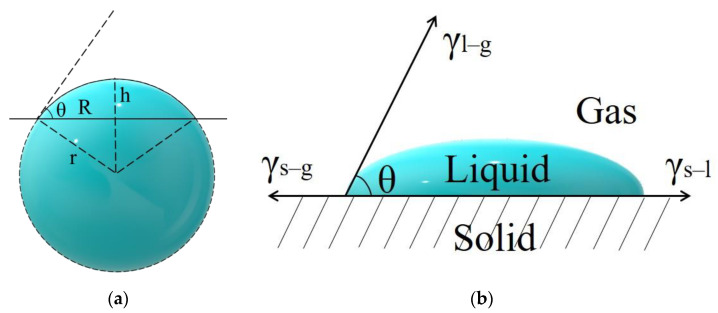
(**a**) Computational model of spherical cap droplet; (**b**) schematic side view of the contact between the droplet and substrate.

Due to the small contact angle of the droplet, it cannot be measured by a contact angle meter. However, the droplet can be regarded as a spherical cap with a volume of 0.5 μL, and then the contact angle can be calculated by measuring the droplet radius *R* (Figure 2a). The calculation formula is

(1) $$V=\frac{\pi h^{2}}{3}\left(3r-h\right)=\frac{\pi h}{6}\left(3R^{2}+h^{2}\right)=0.5$$

(2) $$\theta =\arccos\frac{R^{2}-h^{2}}{R^{2}+h^{2}}$$

The liquid–solid interface wetting of droplets on the substrate can be described by Young’s wetting equation [43] (Figure 2b):

(3) $$\gamma_{s–g}=\gamma_{l–s}+\gamma_{l–g}\cos\theta$$

Therefore, $W_{a}$, $W_{i}$, and  S  can be calculated from the Gibbs free energy change values during the contact transition of the three solid–liquid–gas interfaces when the droplet is in contact with the substrate [44] ($\gamma_{g–l}=-\gamma_{l–g}$):

(4) $$W_{a}=\Delta G=\gamma_{l–s}-\gamma_{l–g}-\gamma_{s–g}=-\gamma_{g–l}\left(1+\cos\theta \right)\;$$

(5) $$W_{i}=\Delta G=\gamma_{l–s}-\gamma_{g–s}=-\gamma_{g–l}\cos\theta$$

(6) $$S=-\Delta G=\gamma_{g–s}-\gamma_{g–l}-\gamma_{l–s}=\gamma_{g–l}\left(\cos\theta -1\right)$$

As can be seen in Table 3, the relationship between solvents for the parameters $\gamma_{g-l}$, θ, $W_{a}$, and S was isopropanol < n-octane < ethanol < ethyl acetate < butyl acetate < acetone < chloroform < toluene. The relationship between solvents for the parameters R and $W_{i}$ was isopropanol > n-octane > ethanol > ethyl acetate > butyl acetate > acetone > chloroform > toluene.

The adhesion of droplets to the substrate increased with the increase in $\left|W_{a}\right|$. The droplets can wet the substrate when $W_{i}\leq 0$, and the wetting ability decreased with the increase in $\left|W_{i}\right|$. The liquid can spread automatically on the substrate when  S≥0, and the spreading ability decreased with the increase in S.

Among the above inks, the θ of inks with isopropanol and n-octane as solvents was no more than 24.86 mN/m. The droplets had weak adhesion and strong wetting and spreading abilities on the substrate due to the small $W_{a}$, and the phenomenon of multilayer ring deposition was more likely to occur. In contrast, the θ and $W_{a}$ of the inks with toluene and chloroform as solvents were higher. Thus, the droplets had stronger adhesion and weaker wetting and spreading abilities on the substrate with no multilayer annular deposition. Therefore, the film-forming property and uniformity can be improved by appropriately increasing the surface tension of the ink.

### 3.2. Effect of Particle Size on Film Morphology

**Figure 3:**
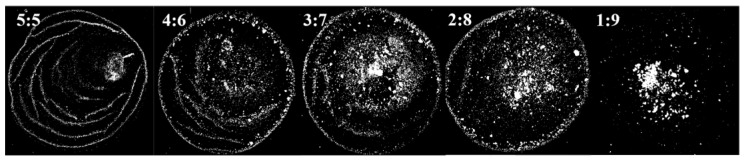
The morphologies of films prepared with different QD inks. The volume ratios of toluene to n-octane in the inks are 5:5, 4:6, 3:7, 2:8, and 1:9.

**Figure 4:**
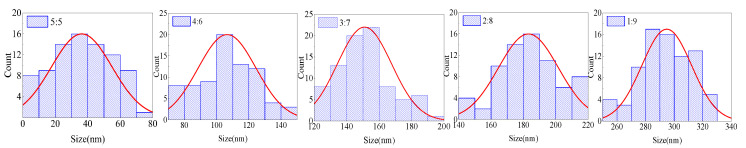
Particle size distribution diagram. The volume ratios of toluene and n-octane in the inks are 5:5, 4:6, 3:7, 2:8, and 1:9.

It was found that the content of the organic solvent in the ink can affect the agglomeration degree of QDs, which in turn affects the morphology of the film. Due to the large difference in polarity, it will cause obvious agglomeration with the addition of n-octane to the QD–toluene solution. Different agglomerated particles can be obtained by adjusting the ratio between n-octane and toluene. Therefore, inks with volume ratios of toluene to n-octane of 5:5, 4:6, 3:7, 2:8, and 1:9 were prepared. The surface tension, particle size, and “coffee ring” width of the films were measured. The film morphology under the microscope is shown in Figure 3. The particle size distribution in the film is shown in Figure 4. The ink surface tension, film particle size, and “coffee ring” width are shown in Table 4.

It can be seen in Table 4 that the surface tension of the ink and the dispersion ability of QDs decreased with the increase in n-octane content. The particle size of the QDs increased, and the “coffee ring” became wider due to agglomeration. It can be seen from the discussion in Section 3.1 that the smaller the surface tension of the ink, the more likely the film has the morphology of multilayer ring deposition. However, the number of “coffee rings” in Figure 3 decreases as the surface tension decreases. This was because the dispersion ability of QDs decreased as the n-octane content increased. It was difficult for the capillary flow in the droplet to push the large particles toward the contact line due to agglomeration. The liquid film evaporated to dryness before the large particles reached the contact line, so the “coffee ring” widened. When the agglomerated particle size of QDs was equal to 188.11 nm, the “coffee ring” and multilayer ring deposition essentially disappeared. When the particle size was equal to 303.89 nm, the large-size particles were primarily concentrated in the center of the film. The “coffee ring” and multilayer annular deposition disappeared completely. Therefore, the film-forming property and uniformity can be improved by appropriately increasing the size of the particle.

### 3.3. Effect of Solute Volume Fraction on Film Morphology

**Figure 5:**
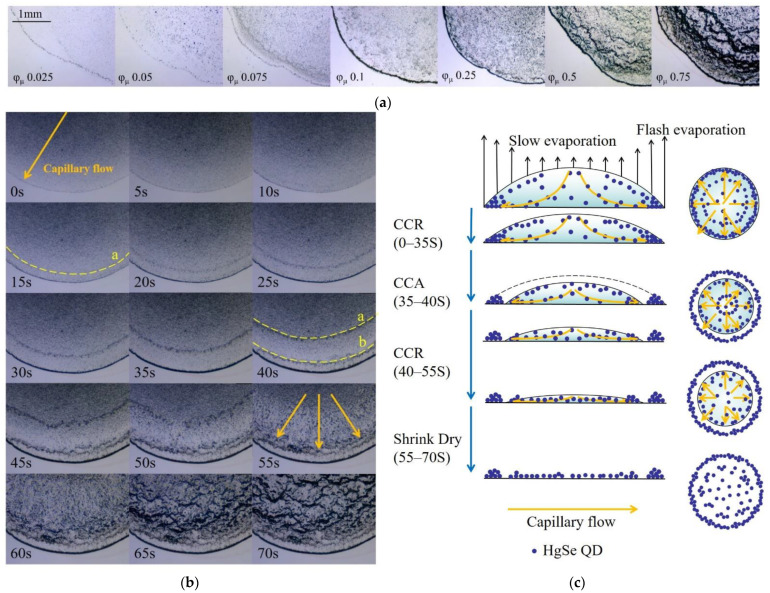
(**a**) The morphologies of films prepared by inks with solute volume fractions of 0.025, 0.05, 0.075, 0.1, 0.25, 0.5, and 0.75 under the microscope. (**b**) The evaporation process when the solute volume fraction was 0.5. (**c**) The schematic side and top views of the liquid film evaporation process when the solute volume fraction was 0.5.

It was found that toluene had the best dispersing effect on QDs. Therefore, toluene was used as the solvent, and QD inks with solute volume fractions of 0.025, 0.05, 0.075, 0.1, 0.25, 0.5, and 0.75 were prepared. The thin films were obtained by evaporation. The morphologies of QD films were observed with an optical microscope, as shown in Figure 5a.

The experimental results show that the solute volume fraction was not the factor that determined the formation of the “coffee ring”. This was only determined by the characteristic of the solvent. Since the solute cannot unpin the contact line and redirect the flow, the evaporation rate of the edge of the liquid film was greater than that of the center when the solvent was constant. In order to keep the contact line pinned, there must be a continuous, radially outward capillary flow from the center to the contact line to compensate for the evaporative removal of the liquid, eventually forming a “coffee ring”.

When the solute volume fraction was less than 0.1, some QDs were deposited on the glass slide before they moved to the edge due to the flash evaporation rate of the solvent and finally formed a uniform QD film. When the solute volume fraction was greater than 0.1 (for example, *φ_μ_* of 0.5), the film evaporation process was more complicated and formed a gully-like morphology, as shown in Figure 5b. The schematic side and top views of the liquid film evaporation process are shown in Figure 5c.

As can be seen in Figure 5b, when the ink contacts the substrate, it forms a liquid film. Then, the three-phase contact line is pinned at once. The QDs in the liquid film began to move to the three-phase contact line. At this time, the evaporation mode was the constant contact radius model (CCR). When the evaporation proceeded for 15 s, the reverse “coffee ring” ‘a’ appeared, gradually widened, and moved toward the center of the liquid film. At the same time, some QDs in ring ‘a’ diffused toward the “coffee ring” under the action of capillary force. The “coffee ring” continued to widen. When the evaporation proceeded for 40 s, the liquid film was released from the pinned “coffee ring”, and the short-term constant contact angle (CCA model) evaporation mode occurred. The pinned ring ‘b’ was the new three-phase contact line, and the film continued to evaporate in CCR mode. As the liquid film gradually became thinner, the temperature difference between the edge and center of the liquid film became smaller, and the moving speed of ring ‘a’ to the center slowed down. When the evaporation progressed to around 70% (at 50 s), ring ‘a’ was fixed and flushed out within 5 s. At the same time, the stably distributed QDs in the center of the liquid film also began to move rapidly to the edge. The liquid film was too thin to be fixed after 15 s, so it shrunk rapidly toward the center and evaporated to dryness. QDs piled up on the edges, eventually forming a “gully” morphology of varying depths. Therefore, in order to avoid the appearance of the “gully” and obtain a more uniform and flat QD film, the volume fraction of the ink solute should not be greater than 0.1.

### 3.4. Infrared Transmittance Analysis of Thin Films

**Figure 6:**
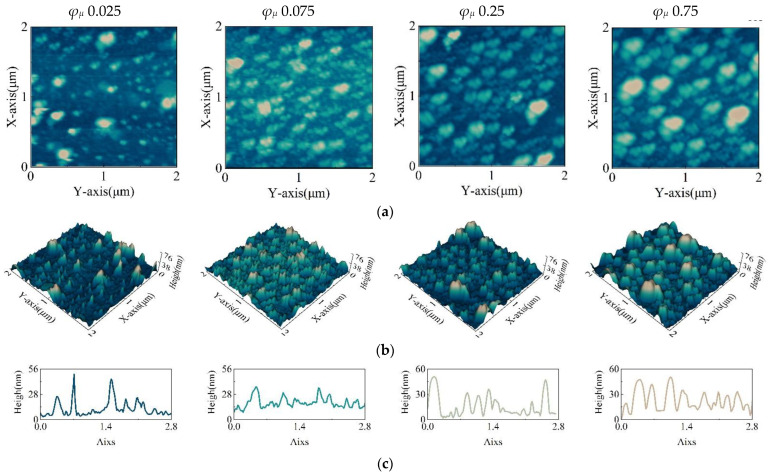
(**a**) Contour map of the film. (**b**) Three-dimensional images of the film under AFM. (**c**) Sectional view of the film’s diagonal. (The volume fractions of ink solute are 0.025, 0.075, 0.25, and 0.75.).

In order to analyze the relationship between film morphology and film transmittance and to provide an empirical method for the subsequent preparation of long-wave infrared QD films, the film morphology was characterized by atomic force microscopy. The 3D surface topography was recorded using a Nanosurf Flex-Axiom atomic force microscope (Nanosurf, AG) in soft tapping mode with a scan speed of 6.25 μm/s to obtain 104 × 104-pixel images. The experiments were carried out at room temperature (297 ± 1 K) using cantilevers with the following nominal properties for force–distance curve measurements: a length of 125 µm, a width of 25 µm, a thickness of 2.1 µm, a tip radius of 10 nm, a force constant of 5 N/m, and a resonance frequency of 150 kHz, as shown in Figure 6.

It can be seen in Figure 6a,b that the QDs are distributed in islands on the substrate. The QDs became denser and higher with the increase in the solute volume fraction. It can be seen in Figure 6c that the shape of the QDs appears broader, and the cross-sectional diameter became larger with the further increase in the solute volume fraction.

**Figure 7:**
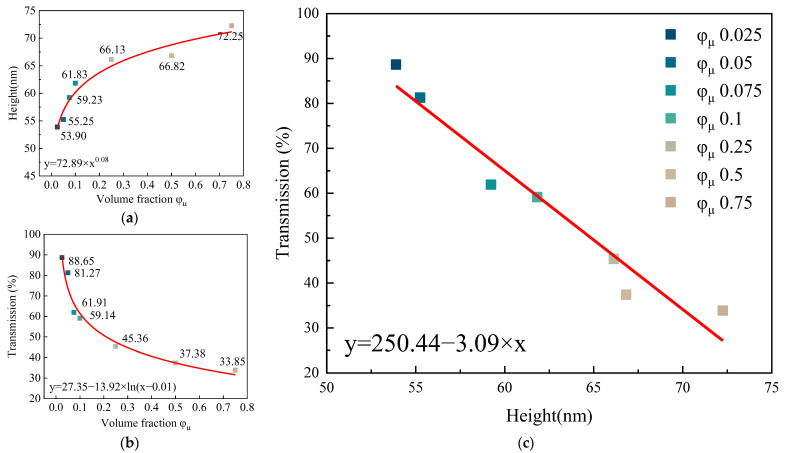
(**a**) Transmittance of films prepared from QD inks with different solute volume fractions. (**b**) Height of films prepared from QD inks with different solute volume fractions. (**c**) The fitting function of solute volume fraction and film transmittance.

The arithmetic mean heights (Sa) of films with solute volume fractions of 0.025, 0.05, 0.075, 0.1, 0.25, 0.5, and 0.75 were 53.90 nm, 55.25 nm, 59.23 nm, 61.83 nm, 66.13 nm, 66.82 nm, ands 72.25 nm, respectively, as shown in Figure 7a. The transmissions of films with solute volume fractions of 0.025, 0.05, 0.075, 0.1, 0.25, 0.5, and 0.75 were 88.65%, 81.27%, 61.91%, 59.14%, 45.36%, 37.38%, and 33.85%, respectively, as shown in Figure 7b. The fitting curve of the film height and transmission is shown in Figure 7c.

It can be seen in Figure 7c that the increase rate of the height of the film and the decrease rate of the transmission at the characteristic peak became slower when the solute volume fraction was 0.1. There was a linear relationship between the height and transmission, which conformed to the Lambert–Beer law. This result can provide an important reference for the preparation of thin films with specific transmission.

## 4. Conclusions

HgSe QD inks with characteristic spectral bands located in the long-wave infrared were prepared by mixing various organic solvents with a toluene solution of QDs. Among them, QD inks with different tensions were first obtained by mixing eight kinds of organic solvents (isopropanol, n-octane, ethanol, ethyl acetate, butyl acetate, acetone, chloroform, and toluene) with the QD–toluene solution. Secondly, due to the large difference in polarity, QD inks with different agglomerated particle sizes were obtained by mixing different proportions of n-octane and QD–toluene solution. QD inks with different solute volume fractions were then prepared by mixing different proportions of toluene and toluene–QD solution. Finally, films with different morphologies were obtained by naturally evaporating QD ink droplets on the substrate. The effects of the surface tension, particle size, and volume fraction on the film morphology were emphasized in the analysis. After that, the infrared transmission spectra of the films were measured. The experimental results suggest that the film slipped in the evaporation process, and the multilayer annular deposition formed when the surface tension of the ink was no more than 24.86 mN/m. The “coffee ring” and the multilayer annular deposition essentially disappeared when the solute particles were larger than 188.11 nm. When the solute volume fraction was greater than 0.1, the QDs in the film were accumulated, and a “gully” morphology appeared. In addition, the increase rate of the film height and the decrease rate of transmission slowed down. The relationship between the film height and transmission was fitted, and the curve conformed to the Lambert–Beer law. Therefore, the morphology of the film can be improved by adjusting the surface tension of the film, the particle size of the solute, and the volume fraction of the solute. Therefore, a uniform and flat film without “coffee rings” can be prepared by adjusting the surface tension, particle size, and volume fraction. This approach could provide an empirical method for the preparation of LWIR QD filter film arrays. It was also found that the evaporation rate, temperature or type of substrate, and shape of solute particles also affected the film morphology in the experiment. The above factors can be discussed and analyzed in detail in subsequent research. In addition, agglomeration easily occurs due to the large specific surface area of nanoparticles. Therefore, it is also important to modify and passivate the surface to avoid agglomeration when synthesizing nanomaterials, and QD filter films with good morphology can be prepared by improving the ink uniformity.

## Figures and Tables

**Table 1:** Existing filter films and their working bands.

Type of QD Filter Array	Operating Range	Reference
CdS, CdSe QD filter array	Vis: 390–690 nm	[13,14]
CdS*_x_*Se_1−*x*_ nanowire filter array	Vis: 500–630 nm	[15]
Perovskite QD filter array	Vis–NIR: 250–1000 nm	[16]
PbS, PbSe QD filter array	NIR: 900–1700 nm	[17,18]

**Table 2:** Sampling amount.

QD Solution (μL)	Toluene (μL)	N-Octane (μL)	Ratio of Toluene to N-Octane
10	40	50	5:5
10	30	60	4:6
10	20	70	3:7
10	10	80	2:8
10	0	90	1:9

**Table 3:** Calculated values of droplet property parameters.

Solvent	Isopropanol	N-Octane	Ethanol	Ethyl Acetate	Butyl Acetate	Acetone	Chloroform	Toluene
Surface tension $\gamma_{g–l}$ (mN/m)	24.10	24.86	25.63	26.53	26.73	27.29	28.02	28.83
Radius R (mm)	2.33	2.17	2.16	2.11	2.07	1.98	1.79	1.62
Contact angle θ (°)	2.89	3.55	3.63	3.88	4.11	4.67	6.38	8.50
*W_a_* (J/m^2^)	48.16	49.68	51.22	52.99	53.39	54.50	55.87	57.35
*W_i_* (J/m^2^)	−24.06	−24.82	−25.58	−26.46	−26.66	−27.20	−27.85	−28.52
*S*	0.03	0.05	0.05	0.06	0.07	0.09	0.17	0.32

**Table 4:** Ink surface tension, particle size, and “coffee ring” width.

Volume Ratio of Toluene to N-Octane	5:5	4:6	3:7	2:8	1:9
Surface tension γ (mN/m)	24.86	24.49	24.36	23.15	22.85
Size (nm)	38.79	106.31	151.78	183.27	294.89
Coffee ring width (μm)	31.41	38.76	50.42	53.23	-

## Data Availability

Not applicable.
